# Knockdown of WHIRLY1 Affects Drought Stress-Induced Leaf Senescence and Histone Modifications of the Senescence-Associated Gene *HvS40*

**DOI:** 10.3390/plants5030037

**Published:** 2016-09-06

**Authors:** Bianka Janack, Paula Sosoi, Karin Krupinska, Klaus Humbeck

**Affiliations:** 1 Institute of Biology, Martin Luther University Halle-Wittenberg, Weinbergweg 10, 06120 Halle, Germany; bianka.janack@pflanzenphys.uni-halle.de; 2 NIRDBS/Stejarul Research Centre for Biological Sciences, Alexandru cel Bun St., 6, 610004 Piatra Neamt, Romania; ppp_paula_2007@yahoo.com; 3 Institute of Botany, Christian-Albrechts-University Kiel, Am Botanischen Garten 1-9, 24098 Kiel, Germany; kkrupinska@bot.uni-kiel.de

**Keywords:** drought stress, epigenetics, histone modification, *HvS40*, leaf senescence, WHIRLY1

## Abstract

The plastid-nucleus located protein WHIRLY1 has been described as an upstream regulator of leaf senescence, binding to the promoter of senescence-associated genes like *HvS40*. To investigate the impact of WHIRLY1 on drought stress-induced, premature senescence, transgenic barley plants with an RNAi-mediated knockdown of the HvWHIRLY1 gene were grown under normal and drought stress conditions. The course of leaf senescence in these lines was monitored by physiological parameters and studies on the expression of senescence- and drought stress-related genes. Drought treatment accelerated leaf senescence in WT plants, whereas WHIRLY 1 knockdown lines (RNAi-W1) showed a stay-green phenotype. Expression of both senescence-associated and drought stress-responsive genes, was delayed in the transgenic plants. Notably, expression of transcription factors of the WRKY and NAC families, which are known to function in senescence- and stress-related signaling pathways, was affected in plants with impaired accumulation of WHIRLY1, indicating that WHIRLY1 acts as an upstream regulator of drought stress-induced senescence. To reveal the epigenetic indexing of *HvS40* at the onset of drought-induced senescence in WT and RNAi-W1 lines, stress-responsive loading with histone modifications of promoter and coding sequences of *HvS40* was analyzed by chromatin immunoprecipitation and quantified by qRT-PCR. In the wildtype, the euchromatic mark H3K9ac of the *HvS40* gene was low under control conditions and was established in response to drought treatment, indicating the action of epigenetic mechanisms in response to drought stress. However, drought stress caused no significant increase in H3K9ac in plants impaired in accumulation of WHIRLY1. The results show that WHIRLY1 knockdown sets in motion a delay in senescence that involves all aspects of gene expression, including changes in chromatin structure.

## 1. Introduction

Leaf senescence is the final step of leaf development, characterized by decreasing photosynthetic activities and yellowing. During leaf senescence, nutritional resources are recycled for re-use in younger leaves and storage organs. In crop plants like barley, orderly progression of leaf senescence is an important determinant of yield. However, adverse environmental conditions, e.g., drought, can cause a premature onset of leaf senescence with inadequate recycling and major losses in yield. Despite the enormous worldwide crop losses by drought stress, the molecular mechanisms underlying drought stress-induced leaf senescence are still unclear.

Both the onset of developmental and stress-induced leaf senescence involves substantial reconstruction of mesophyll cells from sources of photosynthetic assimilates to transport forms of nutrients, generated by the degradation of the compounds of the photosynthetic machinery, in particular proteins [1]. These processes of re-construction are highly regulated and involve massive reprogramming of gene expression [2,3,4,5]. Whereas genes for photosynthetic functions are down-regulated, many genes are up-regulated during senescence and are called senescence-associated genes (SAGs; [5]). In addition, during stress-induced senescence, stress-responsive genes are induced. Many investigations revealed the concerted action of regulatory pathways involving hormones and transcription factors, which are connected to stress and senescence pathways, during transition of a photosynthetically active to a senescing leaf. Recently, it was shown that developmental leaf senescence is under the control of higher-order epigenetic mechanisms, influencing chromatin structure [6,7].

One potential upstream regulator of senescence-associated gene expression is the WHIRLY1 protein. In vitro it was shown to bind to the promoter of the senescence-associated barley gene *HvS40*, the expression of which is also induced by the pathogen *Pyrenophora teres* [8]. Previously, WHIRLY1 was reported to bind to the promoter of the *PR10a* gene in potato [9]. Interestingly, the major part of WHIRLY1 is located in chloroplast nucleoids associated with thylakoid membranes [10]. It has been proposed that upon changes in the redox state of the photosynthetic apparatus, the conformation of WHIRLY1 might be changed to enable translocation to the nucleus [11].

In the present report, we show that WHIRLY1 affects drought stress induction of premature leaf senescence and differential histone modifications of the senescence-associated gene *HvS40*. The results suggest that WHIRLY1 is involved in regulation of drought stress-induced leaf senescence via epigenetic mechanisms.

## 2. Results

### 2.1. WHIRLY1 Acts Upstream of Drought Stress-Induced Leaf Senescence

**Figure 1:**
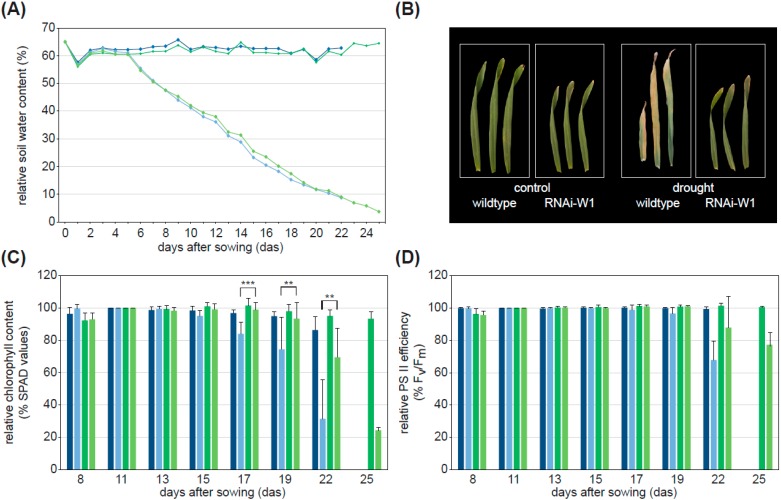
Drought stress-induced differences in the phenotypic appearance and physiological parameters of WT control (blue), WT drought (light blue), RNAi-W1 control (green), RNAi-W1 drought (light green) plants. (**A**) Relative soil water content in control pots and pots drying out with WT and RNAi-W1. To induce drought stress, irrigation was terminated at four das; (**B**) Representative pictures of WT and RNAi-W1 leaves under control and drought-stress conditions at 22 das; (**C**) Chlorophyll content (% SPAD values) and (**D**) PS II efficiency (% F_v_/F_m_) during drought stress in WT and RNAi-W1 leaves compared to controls. Data of 11 das were set as 100%. Asterisks indicate statistically significant differences (paired Student′s *t*-test *p* values, ** *p* < 0.01; *** *p* < 0.001) between drought-stressed WT and RNAi-W1. Data represent the average (± SD) from at least three independent, individual experiments.

Wildtype (WT) and plants of the barley line RNAi-W1, containing only traces of the WHIRLY1 protein [8], were grown under well-watered control and drought stress conditions, respectively. In control pots, the soil water content was kept at 60%. To induce drought stress, irrigation was stopped four days after sowing (das), whereby the relative soil water content was successively decreased (Figure 1A). The drought stress caused premature senescence in WT whereas knockdown of *HvWHIRLY1* reveals a delayed induction of premature senescence under drought conditions. Representative pictures of the barley primary leaves (Figure 1B) and physiological senescence parameters illustrate this. The chlorophyll content of drought-stressed plants started to decrease at 17 das in WT and 19 das in RNAi-W1 plants (Figure 1C). The decline in PS II efficiency began at 19 das in WT and at 22 das in transgenic line (Figure 1D). Plants with a reduced level of WHIRLY1 retained 77% of the starting value of PS II efficiency and 24% of chlorophyll at 25 das, when leaves of drought-stressed WT plants are already dead.

**Figure 2:**
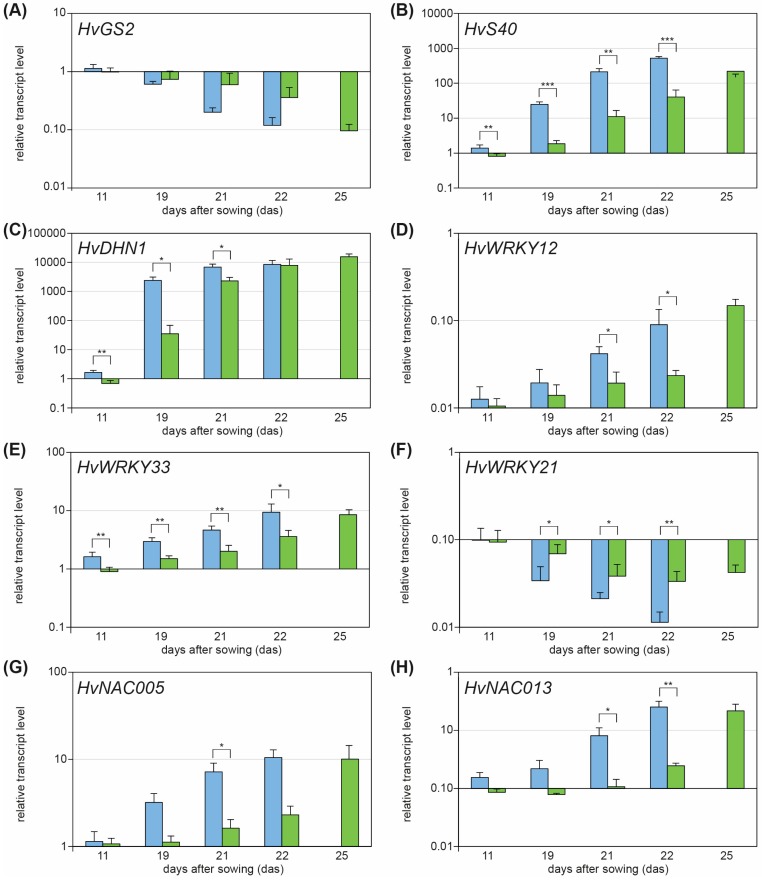
Relative transcript levels in drought-stressed WT (light blue) and RNAi-W1 (light green) plants of (**A**) senescence down-regulated gene *HvGS2*; (**B**) senescence-associated gene *HvS40*; (**C**) drought stress-related *HvDHN1*; (**D**,**E**,**F**) WRKY transcription factor genes *HvWRKY12*, *HvWRKY33* and *HvWRKY21* and (**G**,**H**) NAC transcription factor genes *HvNAC005* and *HvNAC013*. At each data point, transcript levels in drought-stressed plants are compared to those in control plants. Asterisks indicate statistically significant differences (paired Student′s *t*-test *p* values, * *p* < 0.05; ** *p* < 0.01; *** *p* < 0.001) between drought-stressed WT and RNAi-W1. Each data point represents the average (±SD) from at least three independent, individual experiments.

In accordance with the physiological data, drought stress treatment also affected the expression of genes either up- or down-regulated during senescence (Figure 2). *GS2*, encoding the plastid form of the glutamine synthetase, is a typical senescence down-regulated gene [12]. Expression of barley *HvGS2* decreases during developmental senescence of primary leaves [7] but also during drought stress-induced, premature senescence (Figure 2A). *HvS40*, a senescence marker gene induced under dark-induced conditions and during developmental senescence [7,13,14,15], is highly induced during drought stress treatment (Figure 2B). Knockdown of *WHIRLY1* clearly delayed the induction of *HvS40* (Figure 2B). The drought stress treatment also affects the expression of stress-related genes such as *HvDHN1* (encoding a dehydrin of the stress-related LEA protein family; [16,17]). Figure 2C shows that expression of this drought stress marker gene was induced during the stress treatment, but induction was delayed in drought stressed RNAi-W1. Especially WRKY and NAC transcription factors have been described to be involved in stress- and senescence-related pathways [5,18,19,20,21]. While *HvWRKY12* and *HvWRKY33* are induced in response to drought stress, *HvWRKY21* is repressed (Figure 2D–F). As shown in Figure 2G,H the two NAC transcription factor genes *HvNAC005* and *HvNAC013* were also clearly up-regulated in response to drought stress. Induction of these stress- and senescence-related transcription factors, as well as the repression of *HvWRKY21*, was clearly delayed in plants with reduced levels of HvWHIRLY1. These data suggest that HvWHIRLY1 acts as a regulatory factor upstream of drought stress-induced, premature leaf senescence triggered by the concerted action of WRKY and NAC transcription factors.

### 2.2. Knockdown of WHIRLY1 Affected Labeling of HvS40 with Histone Modification Marks

**Figure 3:**
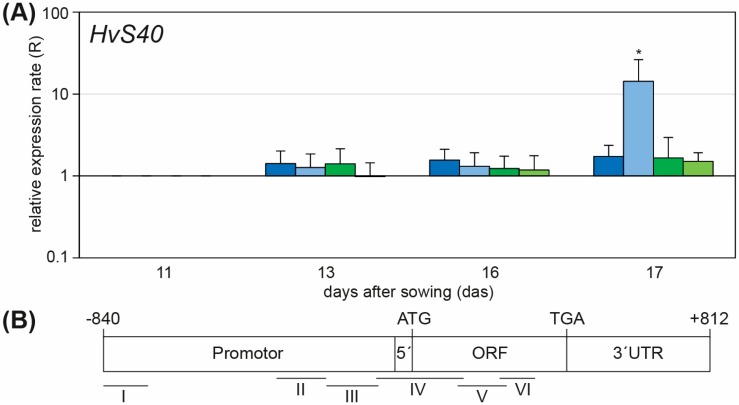

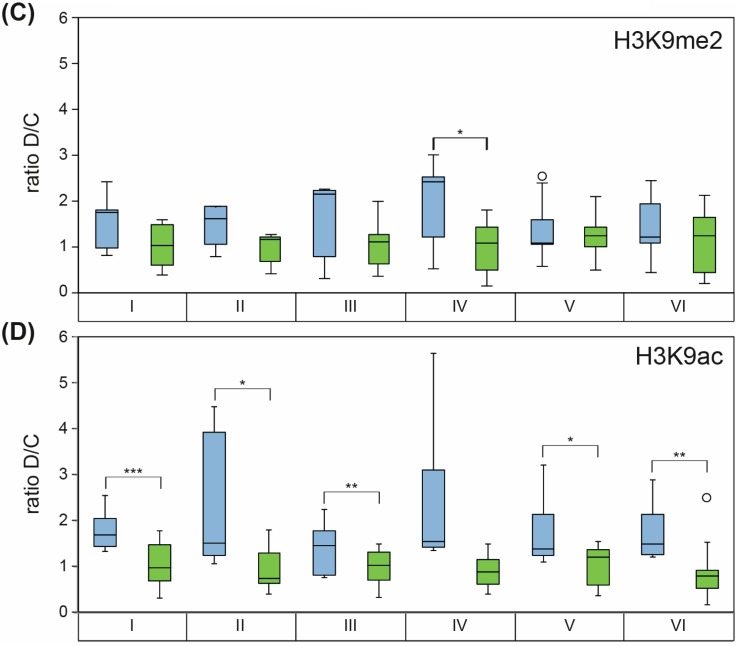
Alterations of histone modification levels of *HvS40* during drought stress-induced senescence of barley primary leaf. (**A**) Relative expression rate of *HvS40* in primary leaves of WT control (blue), WT drought (light blue), RNAi-W1 control (green), RNAi-W1 drought (light green) normalized to the mature leaf (11 das); (**B**) Gene model showing analyzed regions of *HvS40* in promoter (I–III), TLS (IV) and open reading frame (V and VI); (**C**,**D**) Drought stress-specific alterations (ratio drought/control [D/C]) in the level of histone 3 modifications K9me2 and K9ac at *HvS40*, shown as boxplots (central bar marking the median, lower and upper limits of the box marking the 25th and 75th percentiles, and the whiskers extending the 1.5 interquartile range from the box; data not included between the whiskers are dotted as outliers; WT in blue, RNAi-W1 in green). Asterisks indicate statistically significant differences between WT and *RNAi-W1-7* (paired Student’s *t*-test *p* values, * *p* < 0.05; ** *p* < 0.01; *** *p* < 0.001). Each data point represents the average of at least three independent, individual experiments.

Recent investigations showed senescence-specific alterations in histone modifications of senescence-associated genes (SAGs) [6,7]. Senescence and stress are closely linked; thus, drought stress can also induce the expression of several SAGs as displayed for *HvS40* (Figure 2B). This result is in accordance with the high expression of *HvS40* in response to abscisic acid [8]. Analysis under a shorter timescale (Figure 3A) showed that up-regulation of *HvS40* under these conditions started in parallel with the loss of chlorophyll (Figure 1C), at day 17 after sowing in WT plants. To investigate whether the induction of *HvS40* during drought stress is also accompanied by differential histone modifications at its promoter and coding sequence, chromatin immunoprecipitation (ChIP) analyses with antibodies directed against the euchromatic mark H3K9ac (histone 3 lysine 9 acetylation) and the heterochromatic mark H3K9me2 (histone 3 lysine 9 dimethylation), respectively, were performed. Mature leaves of control plants were compared with drought-stressed WT leaves from 17 das, where *HvS40* was not induced in well-watered control plants, but significantly induced in drought-stressed plants (Figure 3A). Quantification of levels of the two markers was performed via qRT-PCR using six primer pairs, covering specific sites in the promoter and coding sequence (positions in relation to the ATG codon I: −840 to −718 bp, II: −369 to −234 bp, III: −232 to −92 bp, IV: −96 to +141 bp, V: +125 to +257 bp and VI: +239 to +334 bp) of *HvS40* (Figure 3B). In Figure 3C, results are plotted for the heterochromatic mark, showing the ratio (D/C) in levels of H3K9me2 in drought-stressed (D) and control (C) leaf samples in WT and RNAi-W1. In addition, in Supplemental Figure S1, levels of labeling with H3K9me2 are separately plotted for control and drought-stressed leaves in both, WT and RNAi-W1. These data show that in WT, drought stress causes a slight increase in H3K9me2 levels of *HvS40*. In RNAi-W1, however, drought stress caused no significant increase in labelling with H3K9me2. In accordance with the induction of *HvS40* (Figure 3A), the euchromatic mark H3K9ac is substantially increased in WT after drought treatment (see Figure 3 and Supplemental Figure S1), resulting in a significant increase in this mark at all promoter and coding sequence regions of *HvS40* analyzed. However, since sonication yields DNA fragments in a range of different sizes, it is difficult to exactly resolve which regions of the gene have H3K9ac-modified histones. In contrast to the WT, in RNAi-W1, H3K9ac levels are not increased (Figure 3D, ratio = 1). This correlates with the finding that, in contrast to the WT, *HvS40* is not induced above the control level in RNAi-W1 plants at 17 das (Figure 3A). Our data revealed that drought stress induces the establishment of euchromatic histone modifications of *HvS40*, and that the function of WHIRLY1 might be connected to the stress and development related setting of epigenetic marks.

## 3. Discussion

Drought stress is a major reason for loss of yield. One consequence of drought stress is premature leaf senescence, which results in early down-regulation of photosynthesis, dismantling of the photosynthetic machinery and inefficient recycling of resources. The molecular response to drought is complex and involves components of abiotic stress response and senescence-associated signaling pathways, e.g., induction of the stress-related LEA gene *HvDHN11* [15,16] and the senescence-associated gene *HvS40* [12,14], the expression of which is highly up-regulated by ABA [8].

Several investigations show that in different plant species, including cereals, specific transcription factors are components of the regulatory pathway of drought stress-induced leaf senescence. In particular, those of the WRKY and NAC families act in this process [22,23,24,25].

Recently, for *Arabidopsis thaliana* [6], but also for barley [7], another superior level of regulation of the senescence program was identified. The authors could show an adjustment of chromatin status of senescence-associated genes via epigenetic mechanisms. It has been demonstrated that especially euchromatic histone modification marks like H3K4me2/3 and H3K9ac are established in respective promoter and coding regions of senescence-associated genes (*AtWRKY53* and *HvS40*), indicating the action of epigenetic mechanisms in regulation of leaf senescence. An increasing number of investigations showed that various types of stress responses are also under the control of epigenetic mechanisms [26,27]. In this study, a differential setting of histone modification marks in response to drought stress at promoter and coding sequences of the senescence-associated barley gene *HvS40* has been demonstrated. Interestingly, in the early response to drought stress, the euchromatic mark H3K9ac was observed to be established at promoter and coding regions of *HvS40*. Establishment of H3K9ac is known to open chromatin structure by changing the charge distribution and thereby allowing access of regulatory proteins to DNA, resulting in induction of transcription [28,29,30,31]. Similar observations have been reported for stress-related genes in *Arabidopsis thaliana* and in rice, where euchromatic marks such as the methylation pattern at H3K4 are set when plants are exposed to drought [32,33,34].

Interestingly, in a recent genome-wide study in *Arabidopsis thaliana*, a positive correlation between gain of H3K4me3 and H3K9ac and the expression of senescence up-regulated genes could be proven [35]. The authors could show an increase in H3K9ac level for Arabidopsis At1g29640, a homolog of barley *HvS40*. After drought treatment a slight, however much less pronounced, increase in H3K9me2 was observed. This is known in *Arabidopsis thaliana* to be associated with constitutive heterochromatic regions [29]. While crop plants in general show higher H3K9me2 levels compared to *Arabidopsis* [36], its function in euchromatic regions is not clearly solved.

In this study, plants with impaired accumulation of WHIRLY1 were shown to have delayed senescence during drought stress. In addition, we can show that impaired accumulation of WHIRLY 1 affects labeling of the senescence-associated gene *HvS40* with the euchromatic mark H3K9ac. Taken together, the results indicate that WHIRLY1 is involved in the signaling pathways during stress-induced senescence acting on epigenetic mechanisms.

WHIRLY1 is a DNA binding protein located in chloroplasts as well as in the nucleus [10] and in transplastomic tobacco plants, was shown to translocate from the organelle to the nucleus [37]. In chloroplasts, it has been identified as a nucleoid-associated protein [38,39,40]. In the nucleus, it has been found in association with promoters of the *PR10a* gene of potato [9] and of *HvS40* [8]. A delay of senescence in response to stress factors in plants with a reduced amount of WHIRLY1 suggests that WHIRLY1 is required for detection of stress, i.e., drought and high irradiance. In *Arabidopsis* seedlings, it has been shown that WHIRLY1 located inside the plastid is required for sensing of exogenously applied salicylic acid and abscisic acid [41]. These hormones are major upstream regulators of pathogen and drought stress responses, respectively [42,43]. Based on these findings, it has been hypothesized that WHIRLY1 located in chloroplast nucleoids bound to thylakoid membranes might act as a sensor and a communicator of hormone-dependent and stress-related redox changes in the photosynthetic apparatus [11].

Although WHIRLY1 has been shown to bind to promoters of *HvS40* and *PR* genes, the molecular mechanism of WHIRLY1 action in the nucleus is not clear. In contrast to conventional transcription factors, WHIRLY1 binds preferentially to single-stranded DNA and RNA [44]. Interestingly, electron microscopy using immunogold labeling revealed that WHIRLY1 in the nucleus was preferentially located in dense, heterochromatic areas [10]. This finding is in accordance with the observation that WHIRLY1 in chloroplasts promotes the compactness of nucleoids [8]. It is hence proposed that WHIRLY1, by interaction with DNA (in plastid and nucleus), affects DNA structure. We hypothesize that WHIRLY1, by interaction with senescence-associated genes, is able to modulate epigenetic mechanisms, which determine local chromatin structure, i.e., histone modifications, DNA methylation and/or chromatin remodeling. This hypothesis has to be tested in future experiments

## 4. Experimental Section

### 4.1. Plant Material and Growth Conditions

Seeds of barley wildtype (*Hordeum vulgare* L. cv Golden Promise) and transgenic barley plants of the line *RNAi-W1*, produced by RNAi-mediated knockdown of the WHIRLY1 gene [45], were germinated on wet tissues for 48 h at 4 °C and 24 h at room temperature in darkness. After germination, barley seedlings (10 plants per pot) were sown in ′Mitscherlich′ pots, each containing 1.3 kg soil (ED73, Einheitserdewerk Hameln A. Stangenberg GmbH, Sinntal, Germany, http://www.einheitserde.de) and were grown in a phytochamber under long-day conditions (16 h with light intensity of 400 µE·m^−2^·s^−1^ at 21 °C and 8 h in the dark at 18 °C, humidity of about 60%). Control plants of wildtype and the RNAi-W1 line were well watered. Their soil water content was kept at 60% throughout plant development. For the drought stress treatment, irrigation was terminated four days after sowing (das), allowing the soil to dry out. All measurements were performed using primary leaves (the first leaf after the coleoptile). For gene expression analysis, complete primary leaf blades from control and treated plants were collected, immediately frozen in liquid nitrogen and stored at −80 °C. For analyzing histone modification patterns by chromatin immunoprecipitation (ChIP), 1 g of total primary leaf blades of each plant line were harvested, fixed with formaldehyde, frozen in liquid nitrogen and stored at −80 °C. Collection of leaf material took place in the middle of the light period. Data were obtained from at least three independent sowings comparing WT and RNAi plants during growth under control and drought conditions.

### 4.2. Physiological Measurements

Measurement of physiological parameters (chlorophyll content and PS II efficiency) of leaf material took place in the middle of the light period. Measured values from 11 das, when primary leaves reached the mature stage of development, were set to 100%.

#### 4.2.1. Chlorophyll Content

Determination of relative chlorophyll content per unit leaf area was carried out by using a SPAD (Soil Plant Analysis Development) analyzer (Konica Minolta Sensing Europe, Munich, Germany, http://www.konicaminolta.eu). Transmittance of red light (650 nm) and infrared (940 nm) radiation is measured noninvasively on intact leaves. Relative SPAD values are related to the chlorophyll content in a linear manner [46]. Each data point represents the average of at least three independent biological replicates with 20 independent measurements at each tip, middle and base of primary leaves.

#### 4.2.2. PS II Efficiency

To determine PS II efficiency (F_v_/F_m_), as a measure of the quantum yield of PS II [47], chlorophyll fluorescence was measured with a Photosynthesis Yield Analyzer (Mini-PAM, Walz GmbH, Effeltrich, Germany, http://www.walz.com) using the pulse amplitude modulation (PAM) method [48]. Measurements were executed at the mid position of 20-min dark-adapted primary leaves. Each data point represents the average of at least three independent biological replicates with 20 independent measurements at the mid position of primary leaves.

### 4.3. Gene Expression

From primary leaves of WT and RNAi-W1 of control and stress-treated plants, total RNA was isolated with trizol as described by Chomczynski and Mackey [49]. By gel electrophoresis (1% TAE agarose gel), integrity of extracted RNA was verified. To prevent contamination by genomic DNA, RNA samples were treated with RNase-free-DNaseI (Roche-Diagnostics GmbH, Mannheim, Germany, www.roche.com/diagnostics). Synthesis of the first-strand cDNA was executed as described in operating manual by reverse transcription of 1 µg of total RNA with RevertAid H Minus Reverse Transcriptase (Thermo Fisher Scientific, Inc. Waltham, MA, USA, www.thermoscientificbio.com) in a volume of 20 μL. qRT-PCR was performed in 20 μL containing 1× Platinum^®^ SYBR^®^ Green qPCR SuperMix-UDG (life technologies™, now part of Thermo Fisher Scientific, Inc. Waltham, MA, USA, www.lifetechnologies.com), 0.2 μM each gene specific primer (see Supplemental Table S1), 10 μM fluorescein (Bio-Rad Laboratories, Inc., Göttingen, Germany, www.biorad.com) for well-factor calibration and 1 μL of diluted (1:4) template cDNA. Primers specific for the *HvACTIN* intron were used to validate the absence of genomic DNA. No-template controls were carried out to exclude the amplification of unspecific products. PCR was executed with MyiQ™2 System from Bio-Rad Laboratories, Inc. (Göttingen, Germany). Relative expression rate of genes of interest were calculated by 2^−ΔΔC^_T_ method [50]. Thereby, expression data of genes of interest (GOI) of control (∆C_Tcontrol_ = C_TGOI_ − C_THvACTIN_) and drought-stressed (∆C_Tdrought_ = C_TGOI_ − C_THvACTIN_) plants of WT and RNAi-W1 were normalized to the reference gene *HvACTIN* expression. In addition, *HvACTIN*, *HvPP2A* was also tested as a reference gene [7] showing similar constant values as *HvActin* (data not shown), but is not as stable in *Hordeum vulgare* L. cv Golden Promise. The values were further normalized to the expression of the gene of interest in control plants at the same time point (2^−(∆C_T_drought − ∆C_T_control)^). Each data point represents the average of measurements of three independent biological replicates.

### 4.4. Chromatin Immunoprecipitation

ChIP was performed as described by Gendrel et al. [51] and Ay et al. [6] with some modifications. From WT and RNAi-W1 control and drought-stressed plants, 1 g of primary leaves were harvested at 17 das and cross-linked (0.4 M sucrose, 10 mM Tris-HCl (pH 8), 1 mM Na-EDTA, 1 mM PMSF and 1% formaldehyde [52]) for 10 min at 4 °C by vacuum infiltration (18–20 mmHg). The cross-linking reaction was then stopped by adding glycine (final concentration of 0.1 M) and another 5 min of vacuum incubation. To extract chromatin, the nuclei were isolated and re-suspended in nuclei lysis buffer (50 mM Tris-HCl (pH 8), 10 mM Na-EDTA, 1% SDS, 0.1 mM PMSF and Protease Inhibitor Cocktail (Roche, Mannheim, Germany). Chromatin was sheared with ultra sound using Bioruptor UCD-200 (Europe-Diagenode, Liége, Belgium, www.diagenode.com) to an average fragment size of 300 bp, which was checked by gel electrophoresis (1% TAE agarose gel) for each biological replicate. Immunoprecipitation was performed overnight at 4 °C with antibodies against H3K9me2 (ab1220) and H3K9ac (ab10812) obtained from Abcam (Cambridge, UK, http://www.abcam.com). In addition, input DNA control (representing total amount of DNA) and no-antibody control (mock) were executed. To isolate and clean immunoprecipitated DNA NucleoSpin® Gel and PCR Clean-up kit (Macherey & Nagel, Düren, Germany, www.mn-net.com) was used. The amount of precipitated DNA was quantified by qRT-PCR. qRT-PCR reactions were performed in a total volume of 20 μL containing 1× Platinum^®^ SYBR^®^ Green qPCR SuperMix-UDG (life technologies™, now part of Thermo Fisher Scientific, Inc. Waltham, MA, USA), 0.2 μM each gene-specific primer (see Supplemental Table S1), 10 μM fluorescein (Bio-Rad Laboratories, Inc., Göttingen, Germany) for well-factor calibration and 5 μL precipitated template DNA. To exclude the amplification of unspecific products, no-template controls were carried out. PCR was executed with the MyiQ™2 System from Bio-Rad. To calculate the percentage input, input DNA control was set to 100% and a standard curve was performed with a dilution series (0.12%, 0.06%, 0.03%, 0.015%, 0.0075%, 0.00375% and 0.001875%) for each gene-specific primer pair. Mock values, which were below 0.1% of input DNA, were subtracted from IP samples. Subsequently, drought-stressed (D) results were divided by the results of untreated mature control (C) primary leaves (ratio D/C) for WT and RNAi-W1, respectively. Each data point represents the average of at least three independent biological replicates.

### 4.5. Primer Design

Primers for expression and chromatin immunoprecipitation analyses were designed by using the PrimerSelectTM tool (Lasergene® from DNAStar, Inc.; Madison, WI, USA, www.dnastar.com). All Primer sequences were verified to be specific for the corresponding gene by using the BLAST tools provided by the National Center for Biotechnology Information (http:// www.ncbi.nlm.nih.gov) and the Leibniz Institute of Plant Genetics and Crop Plant Research (IPK) (http://webblast. ipk-gatersleben.de/barley/).

### 4.6. Accession Number

Sequence data for a *Hordeum vulgare* subsp. vulgare genomic clone of *HvS40* can be found in the GenBank data libraries under accession number FI496079.1.

## 5. Conclusions

WHIRLY1 is an upstream regulator of developmental and drought stress-induced leaf senescence. Its knockdown affects expression of the senescence-associated gene *HvS40*. During drought stress-induced senescence, the euchromatic, transcription activating histone modification H3K9ac is established at promoter and coding sequence of *HvS40* in wildtype, but not in WHIRLY1 knockdown line, indicating WHIRLY1-dependent regulation of drought stress- and senescence-related transcriptional reprogramming via epigenetic mechanisms.

## Supplementary Materials

The following are available online at www.mdpi.com/2223-7747/5/3/37/s1.
