# Supplementary material for: Knockdown of WHIRLY1 Affects Drought Stress-Induced Leaf Senescence and Histone Modifications of the Senescence-Associated Gene HvS40
Source: Plants (Basel). 2016 Sep 6;5(3):37. doi: 10.3390/plants5030037 (PMC5039745; doi:10.3390/plants5030037)
Supplement: Supplementary file 1 [file plants-05-00037-s001.pdf]

Supplementary Materials: Knockdown of WHIRLY1 Affects Drought Stress-Induced Leaf Senescence and Histone Modifications of the Senescence-Associated Gene *HvS40*

Bianka Janack, Paula Sosoi, Karin Krupinska and Klaus Humbeck

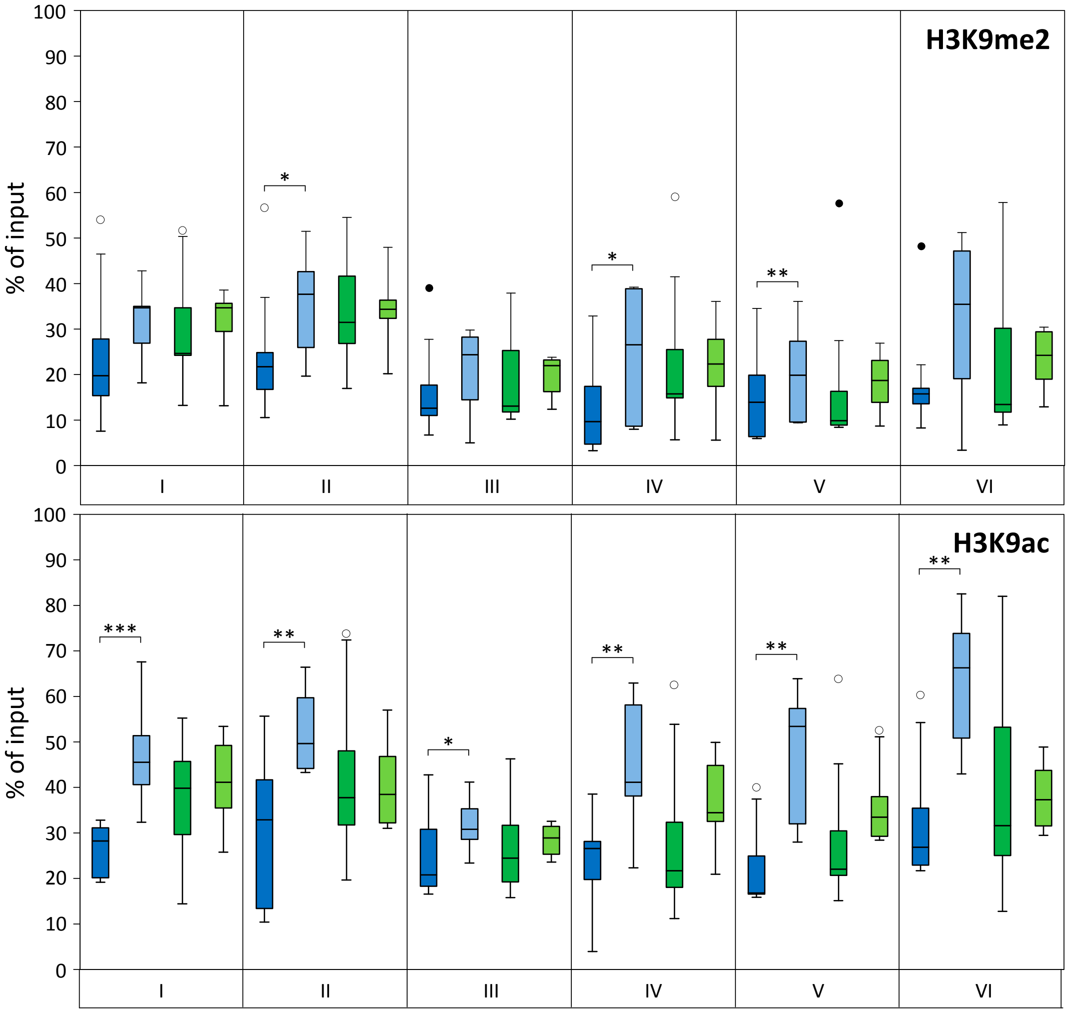

Figure S1. Alterations of histone modification levels (% of input) at *HvS40.* Alterations of histone modification levels at *HvS40* during drought stress-induced senescence of barley primary leaf. Drought stress-specific alterations (% of inputs) in the level of histone 3 modifications K9me2 and K9ac at *HvS40* (promoter (I–III), TLS (IV) and open reading frame (V and VI)) shown as boxplots (central bar marking the median, lower and upper limits of the box marking the 25th and 75th percentiles, and the whiskers extending the 1.5 interquartile range from the box; data not included between the whiskers are dotted as outliers (white dots) and extremes (black dots)). WT control (blue), WT drought stress (light blue), RNAi-W1 control (green), RNAi-W1 drought stress (light green). Asterisks indicate statistically significant differences between control and drought stress (paired Student's t-test p values, * $p < 0.05$; ** $p < 0.01$; *** $p < 0.001$). Each data point represents the average of at least three biological replicates.

Table S1. Oligonucleotide sequences of expression and ChIP analyses.

Gene	Accession Number	Gene Region	Direction	Primer Sequence	Method
HvACTIN	AK365182	intron	forward	5′-AGCTTCGTCCTCATTCTGAGC-3′	qRT-PCR
			reverse	5′-GCGAGGTCAAGACGAAGGAT-3′	
HvACTIN	AY145451	coding region	forward	5′-GGAAATGGCTGACGGTGAGGAC-3′	qRT-PCR
			reverse	5′-GGCGACCAACTATGCTAGGGAAAAC-3′	
HvGS2	AK360336	coding region	forward	5′-ACGAGCGGAGGTTGACAG-3′	qRT-PCR
			reverse	5′-CGCCCCACACGAATAGAGCA-3′	
HvDhn1	AF043087	coding region	forward	5′-GCAACAGATCAGCACACTTCCA-3′	qRT-PCR
			reverse	5′-GCTGACCCTGGTACTCCATTGT-3′	
HvWRKY12	DQ840411	coding region	forward	5′-GTCGTCGTCGTCGGGAAGAAGAAAG-3′	qRT-PCR
			reverse	5′-GGTAGCCGTCGTCGAGGATGTC-3′	
HvWRKY21	DQ863105	coding region	forward	ACTCGCGGTATCTCTAGGGC	qRT-PCR
			reverse	CAGGTCCATCAGTGTCAAAAC	
HvWRKY33	DQ863117	coding region	forward	5′-CCGTGCCGAGCCCCAATCA-3′	qRT-PCR
			reverse	5′-GCGGCGCAAAGGTATCCAC-3′	
HvNAC005	AK251058	coding region	forward	5′-CCATGTGAACAGCAGCGGCAAC-3′	qRT-PCR
			reverse	5′-CCGACGTTGAGGCTGGTGAATC-3′	
HvNAC013	AK251058	coding region	forward	5′-ATGCCGCCGCACATGATGTAC-3′	qRT-PCR
			reverse	5′-ACAGGTCGCCGGAATTAGCG-3	
HvS40	FI496079.1	I (promotor)	forward	5′-CCGCGGGCCTAACCAGAAT-3′	ChIP
			reverse	5′-CTATAAGTCCCACCTAAATCAAACACAAG-3′	
		II (promotor)	forward	5′-CCGCCATTGGTAAGTAGGAC-3′	ChIP
			reverse	5′-GATGGAAGGAAGGAAGGGGTTG-3′	
		III (promotor)	forward	5′-CAGGCGCAGGCAGAGACAC-3′	ChIP
			reverse	5′-GAAGGGGATGGAGATTGGAGAAG-3′	
		IV (transcription start site)	forward	5′-CCTTCCTTCCACAGCACACC-3′	ChIP
			reverse	5′-GAGGACCGGGGCAGACAG-3′	
		V (coding region)	forward	5′-GTCTGCCCCGGTCCTCGTG-3′	qRT-PCR
			reverse	5′-GTTCCTCTTCGCGTCGTTGG-3′	ChIP
		VI (coding region)	forward	5′-CAACGACGCGAAGAGGAAC-3′	ChIP
			reverse	5′-CCGGTGCACATGGAGTAGG-3′	
